# Safety and immunogenicity of ETVAX®, an oral inactivated vaccine against enterotoxigenic *Escherichia coli* diarrhoea: a double-blinded, randomized, placebo-controlled trial amongst Finnish travellers to Benin, West Africa

**DOI:** 10.1093/jtm/taad045

**Published:** 2023-04-26

**Authors:** Anu Kantele, Marianna Riekkinen, T Sakari Jokiranta, Sari H Pakkanen, Jukka-Pekka Pietilä, Anu Patjas, Mari Eriksson, Tamim Khawaja, Peter Klemets, Kati Marttinen, Heli Siikamäki, Anna Lundgren, Jan Holmgren, Agneta Lissmats, Nils Carlin, Ann-Mari Svennerholm

**Affiliations:** Meilahti Vaccine Research Center, MeVac, University of Helsinki and Department of Infectious Diseases, Inflammation Center, HUS, Helsinki University Hospital, Helsinki, Finland; Human Microbiome Research Unit, University of Helsinki, Helsinki, Finland; Travel Clinic, Aava Medical Center, Helsinki, Finland; Meilahti Vaccine Research Center, MeVac, University of Helsinki and Department of Infectious Diseases, Inflammation Center, HUS, Helsinki University Hospital, Helsinki, Finland; Human Microbiome Research Unit, University of Helsinki, Helsinki, Finland; Travel Clinic, Aava Medical Center, Helsinki, Finland; United Medix Laboratories/Synlab Finland Ltd, Helsinki, Finland; Medicum, Department of Bacteriology and Immunology, University of Helsinki, Helsinki, Finland; Mobidiag Ltd, Espoo, Finland; Meilahti Vaccine Research Center, MeVac, University of Helsinki and Department of Infectious Diseases, Inflammation Center, HUS, Helsinki University Hospital, Helsinki, Finland; Human Microbiome Research Unit, University of Helsinki, Helsinki, Finland; Meilahti Vaccine Research Center, MeVac, University of Helsinki and Department of Infectious Diseases, Inflammation Center, HUS, Helsinki University Hospital, Helsinki, Finland; Human Microbiome Research Unit, University of Helsinki, Helsinki, Finland; Travel Clinic, Aava Medical Center, Helsinki, Finland; Meilahti Vaccine Research Center, MeVac, University of Helsinki and Department of Infectious Diseases, Inflammation Center, HUS, Helsinki University Hospital, Helsinki, Finland; Human Microbiome Research Unit, University of Helsinki, Helsinki, Finland; Travel Clinic, Aava Medical Center, Helsinki, Finland; Meilahti Vaccine Research Center, MeVac, University of Helsinki and Department of Infectious Diseases, Inflammation Center, HUS, Helsinki University Hospital, Helsinki, Finland; Meilahti Vaccine Research Center, MeVac, University of Helsinki and Department of Infectious Diseases, Inflammation Center, HUS, Helsinki University Hospital, Helsinki, Finland; Human Microbiome Research Unit, University of Helsinki, Helsinki, Finland; Meilahti Vaccine Research Center, MeVac, University of Helsinki and Department of Infectious Diseases, Inflammation Center, HUS, Helsinki University Hospital, Helsinki, Finland; Meilahti Vaccine Research Center, MeVac, University of Helsinki and Department of Infectious Diseases, Inflammation Center, HUS, Helsinki University Hospital, Helsinki, Finland; Meilahti Vaccine Research Center, MeVac, University of Helsinki and Department of Infectious Diseases, Inflammation Center, HUS, Helsinki University Hospital, Helsinki, Finland; Gothenburg University Vaccine Research Institute, Department of Microbiology and Immunology, Institute of Biomedicine, University of Gothenburg, Gothenburg, Sweden; Gothenburg University Vaccine Research Institute, Department of Microbiology and Immunology, Institute of Biomedicine, University of Gothenburg, Gothenburg, Sweden; Scandinavian Biopharma, Stockholm, Sweden; Scandinavian Biopharma, Stockholm, Sweden; Gothenburg University Vaccine Research Institute, Department of Microbiology and Immunology, Institute of Biomedicine, University of Gothenburg, Gothenburg, Sweden

**Keywords:** Enterotoxigenic *E. coli* (ETEC), travellers’ diarrhoea (TD), ETVAX®, safety, immunogenicity

## Abstract

**Background:**

No licensed human vaccines are available against enterotoxigenic *Escherichia coli* (ETEC), a major diarrhoeal pathogen affecting children in low- and middle-income countries and foreign travellers alike. ETVAX®, a multivalent oral whole-cell vaccine containing four inactivated ETEC strains and the heat-labile enterotoxin B subunit (LTB), has proved promising in Phase 1 and Phase 1/ 2 studies.

**Methods:**

We conducted a Phase 2b double-blinded, randomized, placebo-controlled trial amongst Finnish travellers to Benin, West Africa. This report presents study design and safety and immunogenicity data. Volunteers aged 18–65 years were randomized 1:1 to receive ETVAX® or placebo. They visited Benin for 12 days, provided stool and blood samples and completed adverse event (AE) forms. IgA and IgG antibodies to LTB and O78 lipopolysaccharide (LPS) were measured by electrochemiluminescence.

**Results:**

The AEs did not differ significantly between vaccine (*n* = 374) and placebo (*n* = 375) recipients. Of the solicited AEs, loose stools/diarrhoea (26.7/25.9%) and stomach ache (23.0/20.0%) were reported most commonly. Of all possibly/probably vaccine-related AEs, the most frequent were gastrointestinal symptoms (54.0/48.8%) and nervous system disorders (20.3/25.1%). Serious AEs were recorded for 4.3/5.6%, all unlikely to be vaccine related. Amongst the ETVAX® recipients, LTB-specific IgA antibodies increased 22-fold. For the 370/372 vaccine/placebo recipients, the frequency of ≥2-fold increases against LTB was 81/2.4%, and against O78 LPS 69/2.7%. The majority of ETVAX® recipients (93%) responded to either LTB or O78.

**Conclusions:**

This Phase 2b trial is the largest on ETVAX® undertaken amongst travellers to date. ETVAX® showed an excellent safety profile and proved strongly immunogenic, which encourages the further development of this vaccine.

## Introduction

In low- and middle-income countries (LMICs), diarrhoeal diseases remain a major health problem and one of the most frequent causes of death amongst children under 5 years.^1^ In these regions, one of the most common diarrhoeal agents is enterotoxigenic *Escherichia coli* (ETEC).^2–6^ It has been estimated that without vaccines, ETEC diarrhoea would account for over 13.7 million stunted children and 240 000 child deaths over the 2025–34 period.^7^ ETEC is also one of the three diarrhoeal agents most frequently contracted by visitors to LMICs.^8–12^ There are several vaccines against ETEC in the pipeline, but none licensed thus far.^13^ The oral inactivated multivalent vaccine ETVAX® stands out as the most advanced of the candidates.^14^^,^^15^

At the beginning of an ETEC infection, the bacteria attach to the intestinal epithelium by means of their surface colonization factors (CFs) and start to multiply and produce either a cholera toxin (CT)-like heat-labile toxin (LT) or a heat-stable peptide toxin (ST) or both.^2^ Because of the CFs’ large number and two kinds of toxin, there is a wide variety of human pathogenic ETEC bacteria.^16^ However, as some ETEC types are much more frequent than others, multivalent vaccines containing a few of the most prevalent CFs and an LT ‘toxoid’ antigen may cover ~80% of all ETEC strains.^16–18^ The only ETEC strains not encompassed would thus be those with less common CFs and ST or ST alone.

Immune protection against ETEC diarrhoea is considered to be mainly mediated by locally produced secretory IgA (S-IgA) antibodies against CFs and LT. These antibodies can prevent diarrhoea by inhibiting intestinal bacterial colonization and toxin action.^19–22^ Therefore, to effectively stimulate these intestinal S-IgA immune responses, ETEC vaccine development centres around orally administered formulations containing CF- and LT-derived antigens.^15^^,^^21–23^ Similar immunological mechanisms have been ascribed to the protective efficacy of Dukoral®, a widely licensed whole-cell/cholera toxin B-subunit (CTB) oral cholera vaccine. Because of immunological cross-reactivity between CTB and LT, Dukoral® can confer short-term protection against LT ETEC diarrhoea; in a few countries, therefore, Dukoral® is licensed for prevention against both cholera and ETEC diarrhoea.^24^

The most advanced oral ETEC vaccine candidate ETVAX® vaccine consists of three components: (i) a mixture of four inactivated recombinant *E. coli* strains over-expressing the most prevalent ETEC CF antigens (CFA/I, CS3, CS5 and CS6), (ii) the LCTBA protein antigen that is a chimaera between the LTB and the CTB^25^ and (iii) the double-mutated LT (dmLT) adjuvant.^16^^,^^26^^,^^27^ ETVAX® and the same vaccine without dmLT and with a higher dose dmLT (25 μg) have been tested for safety and immunogenicity in a Phase 1 study amongst 95 healthy adult Swedish volunteers,^28^ 35 of whom were 13–23 months later given a booster dose of vaccine without dmLT.^29^ The safety and immunogenicity of ETVAX® have also been investigated amongst healthy local children and adults in a Phase 1/Phase 2 trial in Bangladesh.^30^^,^^31^ Both studies suggest that ETVAX® is safe and induces strong serum and intestinal-mucosal IgA antibody responses to each of the CF antigens and LTB.

Spurred on by the successful preceding research, we undertook OEV123, a Phase 2b double-blind, randomized controlled trial to study the safety, immunogenicity, diagnostic laboratory tools and efficacy of ETVAX® vaccine amongst a large group of Finnish travellers visiting Benin, West Africa. The present paper describes our research design, results of safety and tolerability as well as data on immunogenicity.

## Materials and Methods

### Study design

OEV123 was a double-blinded, randomized placebo-controlled trial conducted between 31 May 2017 and 15 April 2019 (recruitment 31 May 2017 to 7 February 2019; follow-up until 15 April 2019) at Meilahti Vaccine Research Center, MeVac, HUS Helsinki University Hospital and University of Helsinki, and at two other centres, the Aava Travel Clinic, Helsinki and Villa Karo Research Center, VKRC, in Grand Popo, Benin, West Africa. The volunteers did not receive any payment, but their travel expenses were partly subsidized by the sponsor. Every 2 weeks a new group departed to Benin, the group sizes varying according to the volunteers’ availability. The protocol was approved by the Ethics Committee of HUS (HUS/2231/2016) and the Finnish Medicines Agency (EudraCT 2016-002690-35). The study was recorded in ClinicalTrials.gov under the identifier NCT03729219. All participants provided written informed consent.

**Figure 1:**
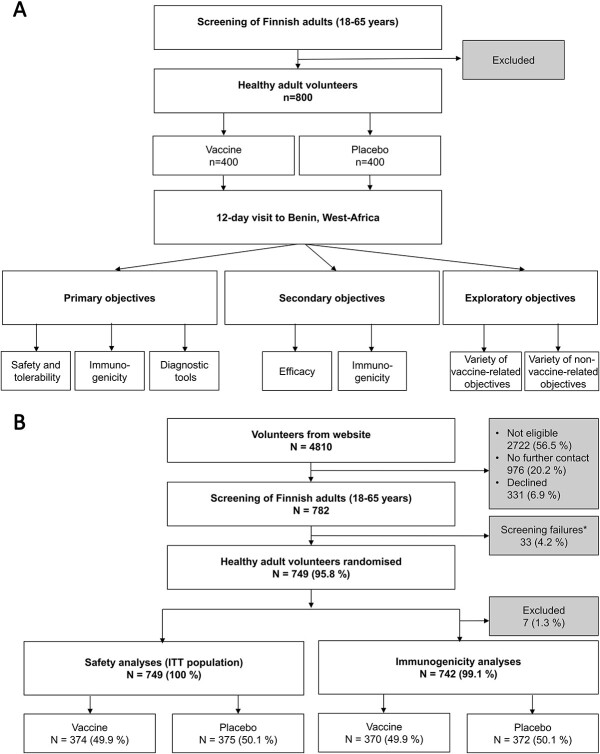
Flow chart of the study conduct of the double-blinded randomized controlled trial on ETVAX®, a multivalent oral vaccine against ETEC, amongst Finnish travellers visiting Grand Popo, Benin, West Africa. (A) Summary of initial study design and objective; (B) flow chart of data presented in the present report. *Main reasons for screening failures: 26/33 (76%) changed their mind after providing written informed consent and 3/33(9%) realized that they would not, after all, be available for all the planned follow-up visits.

Finnish adults (18–65 years of age) were randomized 1:1 to receive ETVAX® or placebo (computed block randomization within the eCRF system in groups of six). The volunteers were asked to provide stool and blood samples and fill in health cards and questionnaires at given time points before, during and after their 12-day visit to Benin (Figure 1 and Supplementary Table 2). As the vaccine needed to be reconstituted on site, the research team included separate unblinded and blinded personnel.

The primary objectives of the OEV123 study were to assess the safety and immunogenicity of ETVAX® and to elucidate the difference between culture-based and non-culture-based diagnostic tools in identification of ETEC. The secondary objectives were to evaluate both efficacy and immunological markers of protection, and to further explore the laboratory tools.

To meet additional exploratory objectives, we expanded our core study OEV123 by conducting further sampling and collecting information by questionnaires (those data are not included here). As a change to the initial study design with 800 volunteers, the recruitment was discontinued slightly before reaching this number. We made the decision because of resource constraints and a far greater diarrhoea incidence than anticipated in the initial power calculations.

### Recruitment, study visits, vaccinations, data collection and sampling

The trial was advertised in newspapers and electronic media with a link to the study website, where those interested were encouraged to fill in a contact request. The Finnish media also published several news releases and interviews on TV and the internet, and in newspapers. The nurses called all those who had left a contact request, provided them with further information and conducted a preliminary screening for eligibility. The pre-screened candidates then attended visit V0 for eligibility assessment and informed consent. For inclusion in the study, the volunteers had to: be ≥18 and ≤65 years of age, agree to regular intake of atovaquone + proguanil (Malarone®) as antimalarial prophylaxis according to prescription guidelines, not have travelled to ETEC endemic areas over the past year and not have taken Dukoral or other cholera or ETEC vaccines within 3 years. Detailed inclusion/exclusion criteria are provided in Supplementary Table 1.

Participation included a pre-travel phase with four visits (V0–V3), a 12-day journey (excluding travel days) to Grand Popo, Benin, with three mandatory visits (B1–B3) and a 1-month post-travel follow-up period with two visits (V4 and V5) (Supplementary Table 2). The vaccine ETVAX® or placebo was administered as a two-dose regimen on visits V1 and V2 14 ± 7 days apart, with a time window allowing the second dose to be given 7–30 days before departure to Benin. Data were collected on adverse event (AE) forms following each vaccine dose (AEF1 and AEF2, see below). Before departure to Benin, blood samples for immunological analyses and routine stool samples for microbial analyses were collected at baseline on V1 before vaccination and at V3, 5–6 days after the second dose.

In Benin, the participants attended three regular visits (B1–B3) and provided a routine stool sample on the fourth day of travel. Visit B1 scheduled within 24 h (or at the maximum 48 h) of arrival included detailed instructions for diarrhoea reporting and stool collection procedures. Travellers’ diarrhoea was defined as passing ≥3 unformed/liquid stools within 24 h together with having one or more of the following gastrointestinal symptoms: fever, abdominal pain, cramping, nausea and vomiting. During each TD episode, the participants delivered samples from the third and fourth stools (if available). They were instructed to contact the subinvestigator at low threshold 24/7 and, if needed, pay an unscheduled visit/ask the subinvestigator for a visit in case of TD or other health issues. During and after their stay in Benin (for 12 days in Benin and 12 days after), the participants recorded their diarrhoeal episodes, assessing severity and reporting related symptoms and concomitant medication on health cards (HC1 and HC2) by hour. Vaccine efficacy calculations were based on episodes occurring between arrival in Benin and 6 days after return to Finland (19 days in all).

The follow-up period in Finland comprised visits V4 on Days 1–6 and V5 on Days 30 ± 5 after return. Routine blood and stool samples were collected on V4 (and V5 for exploratory studies). If TD occurred within 6 days after travel, additional stool samples were collected from the third and fourth stools of the episode.

Data on demographics were collected by questionnaire Q1 before travel. Questionnaire Q2 collected on V4 dealt with travel-related items not covered in HCs. Questionnaire Q3 spanned the period from return to approximately Day 30. Part of Q1 and all of Q2, Q3 and QU4 were devised to meet the exploratory objectives. See Supplementary Table 2 for study procedures.

### ETVAX® vaccine

ETVAX® is a tetravalent ETEC vaccine containing four formaldehyde- or phenol-inactivated recombinant *E. coli* strains, each overexpressing CFA/I, CS3, CS5 or CS6, and a recombinant protein LCTB*A*, a chimaera between heat-labile enterotoxin B subunit (LTB) and CTB-subunit, and dmLT adjuvant. Three of the four vaccine strains have O78 lipopolysaccharide (LPS; same *E. coli* strains were used to overexpress CFA/I, CS3 and CS5). Each full dose of ETVAX® contains 2 × 10^10^ bacteria of each strain, indicating that 6 × 10^10^ bacteria are *E. coli* with O78. The fourth strain (overexpressing CS6) is rough (no O antigen).

Each vaccine vial contains ~8.0 × 10^10^ inactivated bacteria in a volume of 3.3 ml: *E. coli* ETEX 21 containing 0.98 mg CFA/I, *E. coli* ETEX 22 containing 3.13 mg CS3, *E. coli* ETEX23 with 0.63 mg CS5, *E. coli* ETEX24 with 0.145 mg CS6 and, finally, 1 mg of LCTBA, all suspended in phosphate-buffered saline (PBS).

The vaccine vials were produced and released by Cobra Biologics, Matfors, Sweden. The oral dmLT adjuvant LT(R192G/L211A), a genetically modified derivative of wild-type ETEC LT, was produced as a separate lyophilized vial (IDT Biologika GmbH, Germany); the final amount of dmLT was 10 μg/dose.

### Preparation of vaccine and placebo

The vaccine and placebo doses were prepared by unblinded study nurses. For the vaccine group, the content of the dmLT ampoule (700 μg) was reconstituted with 700 μl sterile water and, after dissolving, diluted 1:5 in PBS solution (APL, Sweden) in a 2-ml Eppendorf-tube and refrigerated (2–8°C). The solution was used within 6 h.

Each vaccine dose was prepared by dissolving the sodium bicarbonate (Recipharm AB, Stockholm, Sweden) buffer in 150 ml of cold water and, after dissolving, adding the content of the vaccine vial, followed by 50 μl of dmLT (200 μg/ml) and stirring. The placebo was prepared by dissolving the sodium bicarbonate buffer in 150 ml of cold water. Vaccine and placebo were ingested orally within 30 min after preparation. They were served in white porcelain cups making them indistinguishable from each other, nor was there any obvious dissimilarity in taste.

### Administration of vaccine/placebo regimen

The unblinded study nurses prepared the vaccine/placebo according to the randomization list and the blinded study nurses administered them immediately. Both the vaccine and the placebo were given as a two-dose regimen 14 ± 7 days apart, the first dose at visit V1 and the second at V2. The second dose was given 7–30 days before departure to Benin. A new dose was administered to anyone who vomited within an hour after ingestion of the vaccine/placebo.

**Table 1:** Demographics (ITT population, *n* = 749: 374 in vaccine and 375 in placebo group)

		Total *n* = 749	Active *n* = 374	Placebo *n* = 375
Age, year	Mean (SD)	46.4 (15.6)	46.9 (15.5)	45.9 (15.7)
Height, cm	Mean (SD)	169.9 (8.5)	170.0 (8.5)	169.9 (8.5)
Weight, kg	Mean (SD)	73.7 (15.1)	74.9 (15.5)	72.5 (14.6)
Sex				
	Female, *n* (%)	539 (70.6)	260 (69.5)	269 (71.7)
	Male, *n* (%)	220 (29.4)	114 (30.5)	106 (28.3)
Race				
	Not White or Caucasian, *n* (%)	5 (0.7)	1 (0.3)	4 (1.1)
	White or Caucasian, *n* (%)	743 (99.2)	372 (99.4)	371 (98.9)
	Missing, *n* (%)	1 (0.1)	1 (0.3)	0
Childbearing potential				
	No, *n* (%)	262 (48.6)	139 (53.5)	123 (45.7)
	Yes, *n* (%)	266 (49.4)	120 (46.2)	146 (54.3)
	Missing, *n* (%)	1 (0.2)	1 (0.4)	0
Number of visits to (sub)tropical region				
	Median (min, max)	4 (0, 38)	3 (0, 38)	4 (0, 37)
Time (years) since last visit to (sub) tropics (of those who have travelled^a^)				
	*N*	633	310	323
	Median (min, max)	2 (0, 33)	2 (0, 33)	2 (0, 32)

SD = Standard Deviation

^a^A total of 633/749 participants had travelled to (sub)tropics: 310/374 in active and 323/375 in placebo groups

### Follow-up of AEs

The AE reporting period began upon receipt of the first dose of vaccine/placebo and ended the day the participant travelled to Benin (or at participant’s withdrawal, whichever came first). Ongoing AEs were followed until resolution, the last study visit V5 or participant’s withdrawal, whichever came first. Any trial procedure-related AEs recorded between enrolment and first dose of vaccination or serious adverse events (SAEs) occurring at any time of the study were also reported.

The participants reported solicited and unsolicited AEs for 5 days after each dose on AEF1 and AEF2 forms (active surveillance). Solicited AEs included fever, loose stools/diarrhoea, nausea, stomach ache and vomiting. For the severity grading (mild, moderate and severe) of solicited AEs, see Table 2 and Supplementary Table 3. The severity of each unsolicited AE was evaluated as follows: (i) mild symptoms or signs easily tolerated, not interfering with daily activities (acceptable); (ii) moderate: enough discomfort to interfere with daily activity (disturbing); (iii) severe: prevents daily activities (unacceptable); (iv) life-threatening; (v) fatal.

The relation between AE and vaccine/placebo was categorized as probable, possible or unlikely according to the World Health Organization and Uppsala Monitoring Centre definitions.^32^ SAEs were noted as related or non-related to vaccine/placebo.

**Table 2:** Participants with solicited AEs (ITT population, *n* = 749)

AE^a^	Total *n* = 749, *n* (%)^d^	Active *n* = 374, *n* (%)^d^	Placebo *n* = 375, *n* (%)^d^	Fisher’s exact test *P*-value
At least one solicited AE reported	329 (43.9)	170 (45.5)	159 (42.4)	0.46
**Fever**				0.83
All	22 (2.9)	12 (3.2)	10 (2.7)	
Mild (≥375°C and ≤38.0°C)	16 (72.7)	9 (75.0)	7 (70.0)	
Moderate (>38.0°C and ≤39°C)	6 (27.3)	3 (25.0)	3 (30.0)	
Severe (>39.0°C)	0 (0)	0 (0)	0 (0)	
**Loose stools/diarrhoea** ^b^				0.87
All	197 (26.3)	100 (26.7)	97 (25.9)	
Mild (1–3 episodes of Grade 3–5 stools/24 h)	181 (91.9)	91 (91.0)	90 (92.8)	
Moderate (4–5 episodes of Grade 3–5 stools/24 h)	14 (7.1)	7 (7.0)	7 (7.2)	
Severe (≥6 episodes of Grade 3–5 stools/24 h)	2 (1.0)	2 (2.0)	0 (0)	
**Nausea**				0.24
All	127 (17.0)	70 (18.7)	57 (15.2)	
Mild (does not interfere with normal activities)	97 (76.4)	54 (77.1)	43 (75.4)	
Moderate (interferes with normal activities)	25 (19.7)	14 (20)	11 (19.3)	
Severe (prevents normal activities)	5 (3.9)	2 (2.9)	3 (5.3)	
**Stomach ache**				0.37
All	161 (21.5)	86 (23.0)	75 (20.0)	
Mild (does not interfere with normal activities)	130 (80.7)	71 (82.6)	59 (78.7)	
Moderate (interferes with normal activities)	26 (16.1)	13 (15.1)	13 (17.3)	
Severe (prevents normal activities)	5 (3.1)	2 (2.3)	3 (4.0)	
**Vomiting** ^c^				0.07
All	16 (2.1)	12 (3.2)	4 (1.1)	
Mild (1–2 episodes/24 h)	12 (75.0)	8 (66.7)	4 (100)	
Moderate (3–4 episodes/24 h)	2 (12.5)	2 (16.7)	0 (0)	
Severe (≥5 episodes/24 h)	2 (12.5)	2 (16.7)	0 (0)	

^a^Only the episode with maximum severity of each solicited AE is reported for each participant. Severity grading of solicited AEs (mild, moderate and severe) presented in detail in Supplementary Table 3

^b^Grade 3 = soft, nearly liquid, Grade 4 = colourful liquid and Grade 5 = clear, almost colourless liquid (see Supplementary Figure 1 for visual stool grading seen by participants). The numbers (and proportions) of participants whose highest stool grading was of Grade 3 in total and in active or placebo groups were 152 (77.2%), 75 (75.0%), 77 (79.4%); of Grade 4 41 (20.8%), 22 (22.0%), 19 (19.6%) and of Grade 5 4 (2.0%), 3 (3.0%), 1 (1.0%), respectively

^c^Most vomiting episodes in active group (nine participants) occurred on day of second vaccine dose

^d^Rows ‘All’ show proportions (%) of those with given AEs amongst participants in total and in active or placebo groups. Rows ‘Mild, Moderate and Severe’ show proportions (%) of those with given severity amongst all with the specified AE

### Assessment of immune responses in serum

Vaccine-specific LTB and O78 LPS IgA and IgG antibody levels were determined in serum samples collected shortly before onset of immunization (Day 1) and 5–6 days after second dose. The responses were analyzed by an electrochemiluminescence assay, which is based on the Meso Scale Discovery (MSD) technology using plates precoated with LTB or O78 LPS, respectively, as described in detail earlier.^31^^,^^34^

### Sample size calculation

Sample size calculation was primarily based on the possibility to evaluate the secondary efficacy objective. In the placebo group, vaccine-preventable outcome incidence was assumed at 5% and protective efficacy at 75%. With 80% power and a significance level of 5%, 337 participants per group would be needed. To allow for withdrawals, 400 participants per group were to be included, as also deemed sufficient for the primary objectives.

### Statistical methods

#### Populations

The intention-to-treat (ITT) population consists of all randomized participants (*n* = 749). The safety analysis set includes all randomized participants who received at least one dose. Since all randomized participants received at least one dose, the ITT population coincides with the safety analysis set.

#### Safety analysis

All reported terms (investigator descriptions) for AEs were coded using the Medical Dictionary for Regulatory Activities version 22.1^33^and summarized with frequencies and percentages by treatment group, system organ classification and preferred term. In addition, Fisher’s exact test was used to compare the AE incidences in the vaccine and placebo groups.

#### Immunogenicity analysis

We included in the immunogenicity analysis all randomized participants who received two doses of vaccine and for whom both pre- and post-vaccination sera were available (*n* = 742). The magnitudes of immune responses were determined as post-immunization antibody levels divided by pre-immunization antibody levels; ≥2-fold increases were regarded as significant responses.^28^^,^^30^ As a more stringent measure ≥ 4-fold rises were also determined.^34^ The frequencies of responders in the vaccine and placebo groups were compared by Fisher’s exact test.

### Role of the funding source

The funder participated in the design of the study and analyses and interpretation of data, but not in data collection. The corresponding author had full access to all the data in the study and final responsibility for the decision to submit for publication.

## Results

### Study population

The final ITT population comprised 749 volunteers with a mean age of 46.4 years (range 18–65 years), 70.6% of whom were females. The active (ETVAX®) and placebo groups were comparable with respect to age, sex, height, weight, race and previous travel history to (sub)tropical regions, i.e. countries endemic for ETEC (Table 1). Every 2 weeks new volunteers departed to Benin, the final group sizes varying between 4 and 38, with an average size of 17.

### Adverse events

The total number of solicited and unsolicited AEs amounted to 1006 amongst vaccine recipients and 952 amongst those given placebo; about 77% were mild in both groups (Supplementary Table 4).

### Solicited AEs

The number of participants reporting at least one solicited AE was 170/374 (45.5%) amongst those given vaccine and 159/375 (42.4%) amongst those receiving placebo, with loose stools/diarrhoea (26.7/25.9%) and stomach ache (23.0/20.0%) as the most frequent symptoms, respectively; the vast majority of AEs were reported as mild (Table 2). The most common combination, loose stools/diarrhoea and stomach ache, was reported by 42 (11.2%) in the vaccine and 38 (10.1%) in the placebo group. Vomiting appeared slightly more common amongst those given vaccine than placebo, yet the numbers were low. No statistically significant differences were seen between vaccine and placebo recipients in any of the AEs (Table 2).

As for the timing of AEs in the active group, nausea was mostly reported on the day of vaccine administration: 23 (6.1%) had nausea after the first vaccine dose and 28 (7.5%) after the second. Most vomiting episodes in the active group (nine participants) occurred on the day of the second vaccine dose.

### AEs possibly or probably related to study drug

Of all reported AEs 830/1006 (82.5%) in the active and 751/952 (78.9%) in the placebo group were considered at least possibly related to the study drug (Supplementary Table 5). The proportion of participants with at least one AE possibly or probably related to study drug was 68.4/68.0%, respectively (Table 3). The most commonly reported of these AEs were gastrointestinal disorders (54.0% in active/48.8% in placebo group), nervous system disorders (20.3/25.1%), musculoskeletal and connective tissue disorders (11.8/9.3%), and infections (7.2/6.7%). No statistically significant differences were seen between the two groups (Table 3).

**Table 3:** Participants with AEs possibly or probably related to vaccine/placebo by system organ class (ITT population, *n* = 749)

Body system or organ class of disorder	Total *n* = 749, *n* (%)	Active *n* = 374, *n* (%)	Placebo *n* = 375, *n* (%)	Fisher’s exact test *P*-value
At least one possibly or probably related AE reported	511 (68.2)	256 (68.4)	255 (68.0)	0.895
Blood/lymphatic system	1 (0.1)	0 (0.0)	1 (0.3)	1.000
Cardiac	1 (0.1)	1 (0.3)	0 (0.0)	0.499
Ear and labyrinth	8 (1.1)	6 (1.6)	2 (0.5)	0.177
Eye	1 (0.1)	0 (0.0)	1 (0.3)	1.000
Gastrointestinal	385 (51.4)	202 (54.0)	183 (48.8)	0.165
General/administration site	90 (12.0)	49 (13.1)	41 (10.9)	0.371
Hepatobiliary	1 (0.1)	1 (0.3)	0 (0.0)	0.499
Infections	52 (6.9)	27 (7.2)	25 (6.7)	0.776
Injury, poisoning and complications	2 (0.3)	1 (0.3)	1 (0.3)	1.000
Investigations	2 (0.3)	0 (0.0)	2 (0.5)	0.499
Metabolism and nutrition disorders	5 (0.7)	3 (0.8)	2 (0.5)	0.686
Musculoskeletal and connective tissue	79 (10.6)	44 (11.8)	35 (9.3)	0.287
Nervous system	170 (22.7)	76 (20.3)	94 (25.0)	0.138
Psychiatric	7 (0.9)	3 (0.8)	4 (1.1)	1.000
Renal and urinary	4 (0.5)	1 (0.3)	3 (0.8)	0.624
Reproductive system and breast	10 (1.3)	4 (1.1)	6 (1.6)	0.752
Respiratory, thoracic and mediastinal	53 (7.1)	28 (7.5)	25 (6.7)	0.672
Skin and subcutaneous tissue disorders	22 (2.9)	10 (2.7)	12 (3.2)	0.829
Vascular disorders	5 (0.7)	3 (0.8)	2 (0.5)	0.686

### Serious adverse events

No SAEs were recorded over the AE reporting period, i.e. before departure to Benin. After this period, 17 SAEs were recorded in the active and 24 in the placebo group, all considered unrelated to the study drug. The rates of at least one SAE amongst vaccine versus placebo recipients were 16/374 (4.3%)/21/375 (5.6%); the most common SAEs were infections (3.5/4.5%). No statistically significant differences were observed in the SAE rates between vaccine and placebo recipients (Table 4).

**Table 4:** Participants with SAEs^a^ by system organ class: (ITT population *n* = 749)

Body system or organ class of disorder	Preferred term	Active *n* = 374, *n* (%)	Placebo *n* = 375, *n* (%)	Fisher’s exact test *P*-value
At least one SAE reported	NA	16 (4.3)	21 (5.6)	0.501
Cardiac				0.499
	Stress cardiomyopathy	1 (0.3)	0 (0)	
Gastrointestinal				1.000
	Diarrhoea	0 (0)	1 (0.3)	
General/administration site				0.217^b^
	At least one	1 (0.3)	5 (1.3)	
	Fatigue	0 (0)	1 (0.3)	
	Pyrexia	1 (0.3)	4 (1.1)	
Hepatobiliary				0.499
	Cholelithiasis	1 (0.3)	0 (0)	
Infections				0.577^c^
	At least one	13 (3.5)	17 (4.5)	
	Bacterial infection	0 (0)	1 (0.3)	
	Erysipelas	1 (0.3)	0 (0)	
	Gastroenteritis	4 (1.1)	4 (1.1)	
	Infection	1 (0.3)	1 (0.3)	
	Influenza	3 (0.8)	1 (0.3)	
	Nasopharyngitis	0 (0.0)	1 (0.3)	
	Pneumonia	2 (0.5)	1 (0.3)	
	Pyelonephritis	1 (0.3)	1 (0.3)	
	*Salmonella* sepsis	1 (0.3)	3 (0.8)	
	Salpingitis	0 (0)	1 (0.3)	
	*Shigella* infection	0 (0)	1 (0.3)	
	Upper respiratory tract infection	0 (0)	1 (0.3)	
	Viral upper respiratory tract infection	0 (0)	1 (0.3)	
Injury, poisoning and complications				0.499
	Joint dislocation	1 (0.3)	0 (0)	
Vascular disorders				1.000
	Deep vein thrombosis	0 (0)	1 (0.3)	

^a^All SAEs occurred only after departure to Benin

^b^Fisher’s exact test for the whole group (general/administration site)

^c^Fisher’s exact test for the whole group (infections)

### AEs leading to study discontinuation

Three AE episodes led to discontinuation, two in the active (bloody diarrhoea and cholelithiasis) and one in the placebo group (tooth infection).

## LTB- and O78 LPS-specific IgA and IgG immune responses

**Figure 2:**
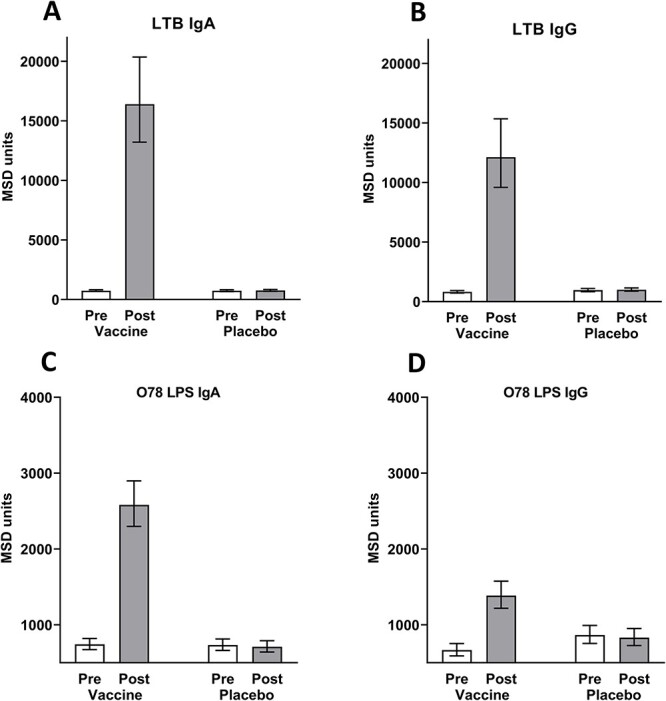
Levels of IgA and IgG antibodies against LTB (A, B) and 078 LPS (C, D) before (pre) and after (post) administration of two doses of ETVAX® or placebo. Antibody levels were measured by MSD electroluminescence and are presented as GM ± 95% CI.

Most of the vaccinees showed a robust LTB-specific immune response after two ETVAX® doses, with GM increasing 22-fold in IgA and 15-fold in IgG antibody levels. Response against O78 LPS was lower: a 3.5-fold GM increase in IgA and 2.1-fold in IgG. The placebo recipients showed no significant increase in the mean antibody levels against either LTB or O78 LPS; the response magnitudes and frequencies are shown in Figure 2 and Table 5, respectively. Of the vaccinees, 93% responded with ≥2-fold increase in levels of antibodies to LTB or O78 LPS.

## Discussion

This is the first trial to explore ETVAX®, currently the most advanced ETEC vaccine candidate, amongst a large traveller population. Earlier the vaccine had successfully undergone a Phase 1 study amongst a smaller group of Swedish adults,^28^^,^^29^ and a large Phase 1/Phase 2 trial in Bangladesh also involving children down to 6 months of age.^30^^,^^31^^,^^34^ The present report on our Phase 2b double-blinded randomized trial amongst healthy travellers visiting Benin describes study design and results concerning the vaccine’s safety, tolerability and immunogenicity. Microbiological data, diagnostic tools and efficacy against ETEC diarrhoea will be covered in subsequent reports.

### Safety and tolerability

Our data for 749 participants comprising 374 vaccine and 375 placebo recipients demonstrate ETVAX® to have an excellent safety and tolerability profile for travellers. The results accord with a previous study where ETVAX® and the same vaccine without the dmLT or with a higher dose of dmLT adjuvant were administered to a total of 95 Swedish volunteers, 30 receiving the same formulation as used in the present study.^28^ We found no statistically significant differences in AEs or SAEs between the 374 vaccine and 375 placebo recipients. Vomiting appeared slightly more frequent in the vaccine group, but the difference did not reach statistical significance and the overall numbers proved low (12 versus 4 cases); monitoring is also warranted in future studies. It should be noted, however, that the rates of both solicited (42.4%) and possibly or probably study drug-related (68.0%) AEs reported by placebo recipients were considerable, highlighting, indeed, the necessity of undertaking double-blinded randomized trials to evaluate adverse effects of any vaccine.

### Immunogenicity

Our study stands out as the largest thus far exploring the immunogenicity of ETVAX® amongst travellers. We examined LTB and LPS responses not only as indicators of immunogenicity but also as measures of vaccine intake pertinent to vaccine safety. Our results confirm previous findings showing that amongst the great majority of adults ETVAX® induces high IgA and IgG serum antibody responses against LTB.^28^^,^^29^^,^^31^ Data from our present large study population also confirms that the serum IgA antibody responses to ETVAX® are somewhat higher and more frequent than serum IgG responses.

Responses against the O78 LPS antigen present in three of the four ETVAX® strains (fourth one a rough strain) were also frequent, with magnitudes comparable to those seen previously for adult Swedish volunteers as well as children in Bangladesh.^28^^,^^34^ A detailed description of systemic and mucosal immune responses against O78 LPS amongst recipients of ETVAX® has been published earlier.^34^

### Limitations

We did not measure antibody responses in stools or cultures of intestine-derived B cells. While these responses may more directly reflect the intestinal SIgA responses, the workload entailed by such large cohort would have been overwhelming and, indeed, the decision appeared justified, since our previous studies have already shown ETVAX® to elicit robust anti-LTB and anti-LPS IgA responses both in faeces and cultures of intestine-derived peripheral blood B cells.^21^^,^^28^^,^^34^ Nor did we conduct any analyses of anti-CF antibody responses in serum, because our previous studies indicate that, in contrast to the results for adults previously primed by ETEC in endemic regions,^31^ only poor anti-CF responses are elicited by ETVAX® in the sera of naive vaccinees.^28^

The bicarbonate buffer used in vaccine/placebo may account for part of the symptoms in both groups and also explain some of the high AE rates in the placebo group. Indeed, our analysis comparing the two groups aimed at revealing reactions to the vaccine component in the active preparation.

**Table 5:** Proportion of participants with ≥2-fold (≥4-fold) increase in LTB- and O78 LPS-specific IgA and IgG levels over study period spanning between pre-immunization and post-immunization in vaccine and placebo groups^a^

	Frequencies of responders ≥2-fold (≥4-fold)			
Antigens	Vaccinees IgA *n* = 370	Placebos IgA *n* = 372	Vaccinees IgG *n* = 370	Placebos IgG *n* = 371
LTB	81.4% (75.9%)	2.4% (0%)	73.2% (66.5%)	1.6% (0.5%)
O78 LPS	70.0% (41.9%)	3.0% (0.8%)	44.1% (20.3%)	2.2% (0.3%)
LTB and O78 LPS	58.1% (31.4%)	1.3% (0%)	35.4% (15.7%)	0.3% (0%)
LTB or O78 LPS	93.2% (86.5%)	4.0% (0.8%)	81.9% (71.1%)	3.5% (0.8%)

^a^All differences between vaccine and placebo groups were statistically highly significant

## Conclusion

Exploring ETVAX® for the first time in a large traveller population, the present investigation finds this vaccine to be safe and well tolerated, showing no significant differences in AEs between vaccine and placebo recipients. The study also demonstrates ETVAX® to be highly immunogenic in a traveller population, inducing robust antibacterial and antitoxin immune responses.

## Supplementary Material

JTM_Supplementary_material_ETVAX_safety_manu_050123_taad045
