# Supplementary material for: Safety and immunogenicity of ETVAX®, an oral inactivated vaccine against enterotoxigenic Escherichia coli diarrhoea: a double-blinded, randomized, placebo-controlled trial amongst Finnish travellers to Benin, West Africa
Source: J Travel Med. 2023 Apr 26;30(7):taad045. doi: 10.1093/jtm/taad045 (PMC10658657; doi:10.1093/jtm/taad045)
Supplement: JTM_Supplementary_material_ETVAX_safety_manu_050123_taad045 [file jtm_supplementary_material_etvax_safety_manu_050123_taad045.docx]

Supplementary material

# Safety and immunogenicity of ETVAX®, an oral inactivated vaccine against enterotoxigenic *Escherichia coli* diarrhoea: a double-blinded, randomised, placebo-controlled trial among Finnish travellers to Benin, West-Africa

Table of Contents

Supplementary Table 1 2

Supplementary Table 2 3

Supplementary Table 3 4

Supplementary Table 4 4

Supplementary Table 5 4

Supplementary Figure 1 5

**Supplementary Table 1.** Inclusion and exclusion criteria.

| **Inclusion criteria** |  |
| --- | --- |
|  | Male or female ≥18 and ≤ 65 years of age. |
|  | General good health at the time of first vaccination. |
|  | Female participants of childbearing potential must not be pregnant. |
|  | Females of childbearing potential must agree to use an efficacious hormonal or barrier method of birth control during the study. |
|  | Willingness to participate in the study after all aspects of the protocol have been explained and written informed consent obtained. |
|  | Availability for duration of study, including all planned follow-up visits. |
|  | Intake of atovaquone + proguanil (Malarone®) as anti-malaria prophylaxis according to prescription guidelines mandatory before, during and after travel to Benin. |
| **Exclusion criteria** |  |
|  | A medical or psychiatric condition which the investigator finds significant enough to preclude participation in the study. |
|  | Known or suspected history of drug, chemical or alcohol abuse, as deemed by the investigator/physician; Alcohol Use Disorders Identification Test (AUDIT) > 13 points. |
|  | Known recent history of impaired immune function which the investigator suspects could influence the immune response.  Intention to get any other investigational vaccine during the study period or within two weeks prior to study vaccination. |
|  | Intention to get any other investigational vaccine during the study period or within two weeks prior to study vaccination. |
|  | Intention to donate blood at any time during the study. |
|  | An acute or chronic medical condition that, as judged by the investigator/physician, might render ingestion of the investigational products unsafe or interfere with the evaluation of responses. This includes but is not limited to gastrointestinal diseases and autoimmune diseases requiring treatment. |
|  | Any history of psychosis or bipolar disorder or on-going significant mental disorder. |
|  | Regular (daily) use of laxatives or agents which lower stomach acidity (antacids, proton pump inhibitors) less than one week before visit V1. |
|  | Use of any oral or parenteral medication known to affect the immune function (e.g., corticosteroids and others) within 30 days preceding the first vaccination or planned use during the active study period. |
|  | Travel to ETEC-endemic areas within the last year or visit for > two months in ETEC endemic areas over the past 10 years. |
|  | Receipt of Dukoral or other ETEC or cholera vaccines within 3 years or planned receipt of such vaccine except ETVAX® during the study. |
|  | Antibiotic therapy within two weeks prior to the vaccination. |
|  | History of diarrhoea within seven days prior to vaccination (defined as ≥ 3 unformed loose stools in 24 hours). |
|  | Any other criteria which, in the investigator's opinion, would compromise the traveller’s ability to participate or the safety or results of the study. |

**Supplementary Table 2**. Study procedures.

| **Visit** | **Pre1** | **V0** | **V1** | **V2** | **V3** | **B1** | **D4** | **B2** | **B3** | **V4** | **V5** |
| --- | --- | --- | --- | --- | --- | --- | --- | --- | --- | --- | --- |
| Days |  |  | 0 | 14±7  days from V1 | 5–6  days from  V2 | within 48h upon arrival | 4th day in Benin | 4–8 days from B1 | 1–2 days before departure | 1–6 days after  return | 30±5 days after return |
| Type of visit | Call | Visit=V | V | V | V | V | Stool | V | V | V | V |
| Eligibility | (x) | x | (x) |  |  |  |  |  |  |  |  |
| ICF, study number assignation |  | x |  |  |  |  |  |  |  |  |  |
| Urine pregnancy test |  |  | x |  |  |  |  |  |  |  |  |
| Body temperature |  |  | x | x |  |  |  |  |  |  |  |
| Randomisation |  |  | x |  |  |  |  |  |  |  |  |
| Vaccination (ETVAX® or placebo) |  |  | x | x |  |  |  |  |  |  |  |
| Travel-related vaccinations |  | x |  |  |  |  |  |  |  |  |  |
| Malaria prophylaxis checked |  |  |  |  |  | x |  | x | x | x |  |
| **Samples** |  |  |  |  |  |  |  |  |  |  |  |
| Blood sampling |  |  | x |  | x |  |  |  |  | x | x* |
| Saliva sampling* |  |  | x* | (x*) |  |  |  |  |  |  |  |
| Routine stool sampling |  |  | x |  | x |  | x |  |  | x | x* |
| TD stool sampling |  |  |  |  |  | x** | x** | x** | x** | x** | x** |
| UTI urine sampling* |  |  |  |  |  | x*** | x*** | x*** | x*** | x*** | x*** |
| **Safety** |  |  |  |  |  |  |  |  |  |  |  |
| AE collection |  | x¤ | x | x | x | x |  |  |  |  |  |
| SAE collection |  | x¤ | x | x | x | x | x | x | x | x | x |
| Immediate post- vaccination AEs (15 min) |  |  | x | x |  |  |  |  |  |  |  |
| AEF1 |  |  | D | C |  |  |  |  |  |  |  |
| AEF2 |  |  |  | D | C |  |  |  |  |  |  |
| HC1 |  |  |  |  |  | D |  | R | C |  |  |
| HC2 |  |  |  |  |  |  |  |  | D | R | C |
| Questionnaires |  |  |  |  |  |  |  |  |  |  |  |
| AUDIT |  | x |  |  |  |  |  |  |  |  |  |
| Q1 |  | D | C |  |  |  |  |  |  |  |  |
| Q2* |  |  |  |  |  |  |  |  |  | x* |  |
| Q3* |  |  |  |  |  |  |  |  |  |  | x* |
| QU4* |  |  |  |  |  | x*** | x*** | x*** | x*** | x*** | x*** |

*Collected only for exploratory purposes, not discussed in this manuscript.

**In case of diarrhoea episodes during stay in Benin or 6 days after return to Finland, samples from third and fourth stools were collected.

***Collected only for exploratory purposes from those with UTI symptoms, not discussed in this manuscript.

¤ Only study procedure-related AEs and SAEs were recorded before administration of the first vaccine dose.

Abbreviations: Informed consent form (ICF), Travellers’ diarrhoea (TD), Urinary tract infection (UTI), Adverse event (AE), Serious adverse event (SAE), Adverse event form (AEF), Dispense (D), Review (R), Collection (C), Health card (HC), Alcohol use disorders identification test (AUDIT), Questionnaire (Q)

**Supplementary Table 3.** Severity grading of solicited adverse events (AEs)

| **Type of AE** | **1 - Mild** | **2 - Moderate** | **3 - Severe** |
| --- | --- | --- | --- |
| Nausea and stomach ache | An adverse event which is relatively mild and transient in nature, but can be an annoyance although it *does not interfere with normal activities.* | An adverse event which may be uncomfortable but is not hazardous to health. It may be sufficiently discomforting to *interfere with normal activities but does not completely prevent them.* | An adverse event which is *incapacitating and prevents normal activities and/or might pose a hazard to the participant* |
| Loose stools/diarrhoea* | 1–3 episodes of grade 3–5 stools within a 24-hour period | 4–5 episodes of grade 3–5 stools within a 24-hour period | ≥ 6 episodes of grade 3–5 stools within a 24-hour period |
| Vomiting | 1–2 episodes within a 24-hour period | 3–4 episodes within a 24-hour period | ≥5 episodes within a 24-hour period |
| Fever (oral) | highest value within a 24-hour period ≥37.5°C and ≤38.0°C | highest value within a 24-hour period >38.0°C and ≤39°C | highest value within a 24-hour period >39.0°C |

*See Supplementary Figure 1 for visual stool grading shown to participants.

**Supplementary Table 4.** Number of all adverse events by relationship to study drug (unlikely, possible, probable) among ITT participants

| **Event severity** | **Total**  **n (%)** | **Active**  **n (%)** | **Placebo**  **n (%)** |
| --- | --- | --- | --- |
| Total number of AEs | 1958 | 1006 | 952 |
| Missing value | 5 (0·3) | 4 (0·4) | 1 (0·1) |
| Unlikely* | 372 (19·0) | 172 (17·1) | 200 (21·0) |
| Possible | 714 (36·5) | 375 (37·3) | 339 (35·6) |
| Probable | 867 (44·3) | 455 (45·2) | 412 (43·3) |

*All SAEs were considered unlikely to be related to study drug.

**Supplementary Table 5.** Number of all adverse events by event severity (mild, moderate, severe, life threatening) among ITT participants

| **Event severity** | **Total**  **n (%)** | **Active**  **n (%)** | **Placebo**  **n (%)** |
| --- | --- | --- | --- |
| Total number of AEs | 1958 | 1006 | 952 |
| Missing value | 15 (0·8) | 11 (1·1) | 4 (0·4) |
| Mild | 1511 (77·2) | 773 (76·8) | 738 (77·5) |
| Moderate | 388 (19·8) | 202 (20·1) | 186 (19·5) |
| Severe | 43 (2·2) | 19 (1·9) | 24 (2·5) |
| Life-threatening | 1 (0·05) | 1 (0·1)* | 0 (0) |

*SAE, unlikely to be related to study drug.

**Supplementary Figure 1**. Visual stool grading seen by participants when evaluating loose stools/diarrhoea on AEF1 and AEF2


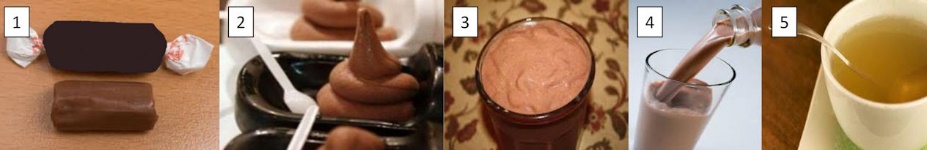


Grade 1 = Firm; grade 2 = Soft, formed; grade 3 = Soft, nearly liquid; grade 4 = Colourful liquid; grade 5 = Clear, almost colourless liquid
